# Serum-dependent processing of late apoptotic cells for enhanced efferocytosis

**DOI:** 10.1038/cddis.2014.210

**Published:** 2014-05-29

**Authors:** Y Y Liang, T Arnold, A Michlmayr, D Rainprecht, B Perticevic, A Spittler, R Oehler

**Affiliations:** 1 Department of Surgery, Surgical Research Laboratories, Comprehensive Cancer Center, Medical University of Vienna, Vienna, Austria

**Keywords:** complement component C1q, phagocytosis, serum, efferocytosis

## Abstract

Binding of the serum protein complement component C1q to the surface of dying cells facilitates their clearance by phagocytes in a process termed efferocytosis. Here, we investigate during which phase of apoptotic cell death progression C1q binding takes place. Purified C1q was found to bind to all dying cells and, albeit weaker, also to viable cells. The presence of serum abrogated completely the binding to viable cells. In addition, C1q binding to dying cells was limited to a specific subpopulation of late apoptotic/secondary necrotic cells. Co-culturing serum-treated apoptotic cells with human monocytes revealed a much higher phagocytosis of C1q-positive than of C1q-negative late apoptotic/secondary necrotic cells. But this phagocytosis-promoting activity could not be observed with purified C1q. Serum-treated C1q-positive late apoptotic/secondary necrotic cells exhibited a similar volume, a similar degraded protein composition, but a much lower DNA content in comparison with the remaining late apoptotic/secondary necrotic cells. This was mediated by a serum-bound nuclease activity that could be abrogated by G-actin, which is a specific inhibitor of serum DNase I. These results show that serum factors are involved in the prevention of C1q binding to viable cells and in the processing of late apoptotic/secondary necrotic cells promoting cell death progression toward apoptotic bodies. This process leads to the exposure of C1q-binding structures and facilitates efferocytosis.

Efferocytosis (i.e., the removal of apoptotic or necrotic cells) is essential for tissue homeostasis and resolution of inflammation.^1, 2^ Early apoptotic cells release a number of ‘find-me' signals to attract phagocytes without induction of inflammation.^3^ Late apoptotic/secondary necrotic cells as well as primary necrotic cells release additional pro-inflammatory ‘danger signals' such as HMGB1.^4, 5, 6^ Furthermore, dying cells expose ‘eat me' signals such as phosphatidylserine, oxidized phospholipids, nucleic acids and histones. Phagocytes bind either directly to such structures or indirectly involving so-called ‘bridging factors'. These molecules derive from serum or interstitial fluid and serve as links between the phagocyte and its prey. The best characterized bridging molecule is the complement component C1q.^7, 8, 9, 10^ Accordingly, inherited deficiency in C1q leads to an impaired clearance of apoptotic cells and is strongly associated with the development of autoimmunity.^11, 12, 13^ The reduced efferocytosis can be restored when C1q is substituted.^12^ Also other complement activating molecules (MBL, ficolins, natural antibodies and surfactant proteins), C-reactive protein, annexins and thrombospondin-1 have been described to act as bridging factors.^4, 14^ In addition, serum-derived complement regulators,^15, 16^ DNases^17^ and proteases^18^ were shown to be involved in the process of efferocytosis. Efficient efferocytosis is particularly required during chemotherapy, which induces massive apoptosis of tumor cells and is frequently associated with leukopenia. In a previous proteomic analysis of breast cancer patients responding to an epirubicin/docetaxel combination therapy, we found significant changes in the plasma levels of C1q and of several activation fragments of C3 and C4.^19^ In addition, an increase of HMGB1 was observed.^20^ In non-responders, however, the complement system as well as HMGB1 remained unaffected. This indicates that successful chemotherapy results in apoptotic tumor cells, which are then opsonized by the complement system.

Apoptotic cell death progression includes chromatin condensation, nuclear fragmentation, plasma membrane blebbing, cellular shrinkage, cell membrane disintegration and results finally in the formation of cellular remnants.^21, 22^ The present study investigates during which phase of apoptotic cell death progression C1q binding takes place. We could show that C1q binds exclusively to a subpopulation of late apoptotic/secondary necrotic cells, which is formed by a serum-bound DNase activity. This processing was found to be essential for the efferocytosis-promoting activity of C1q.

## Results

### Binding of C1q to dying cells

Different chemotherapeutic drugs (oxaliplatin, irinotecan, etoposide and 5-FU) or UV-C irradiation was used to induce apoptosis in Jurkat cells. Apoptotic cell death was confirmed in time course and titration experiments by morphological (cell shrinkage and formation of apoptotic blebs) as well as by molecular parameters (zVAD-sensitive activation of caspases 8 and 3 and binding of annexin A5; see Supplementary Figures 1 and 2). For all further experiments, we chose conditions that reduced the cell viability by 70% within 48 h. At this time point, around 50% of all cells were permeable for PI indicating that they were already in the late apoptotic/secondary necrotic phase. In control experiments, we induced primary necrosis by heat treatment (58 °C for 20 min) that resulted in immediate cell swelling and increased cell membrane permeability in the absence of any caspase-3 activation (Supplementary Figure 2).

**Figure 1:**
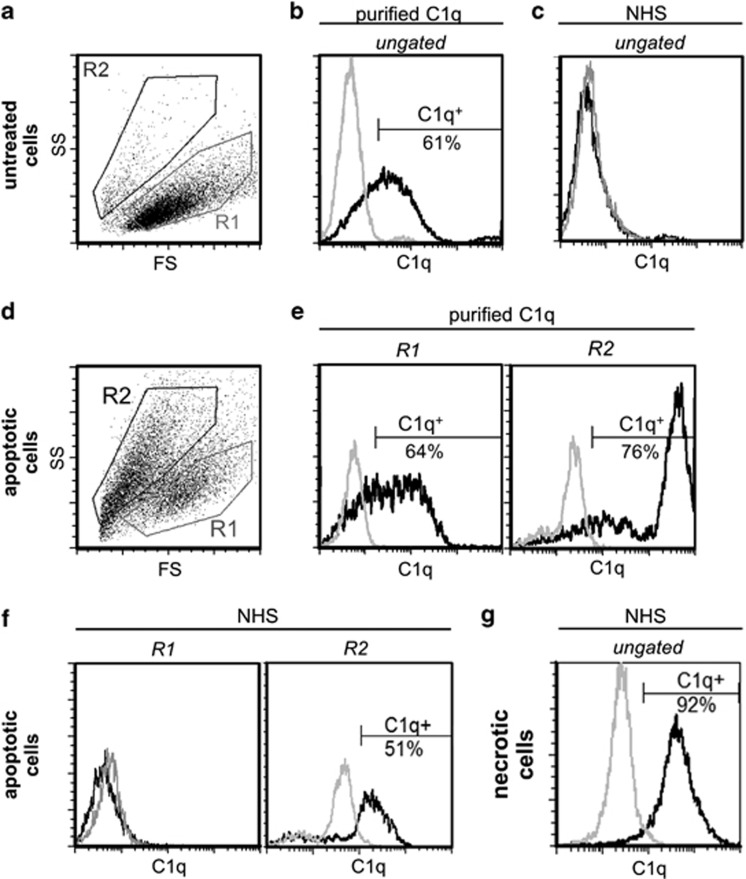
C1q binding to dying cells. Jurkat cells were cultured in UC medium in the absence (**a**–**c**) or presence (**d**–**f**) of 20 *μ*M oxaliplatin for 48 h for induction of apoptosis. Then cells were incubated for an additional hour either in UC medium containing 20 *μ*g/ml purified C1q (**b** and **e**) or in UC medium supplemented with 25% NHS (**c** and **f**). Primary necrotic cells (**g**) were incubated for an additional hour in UC medium supplemented with 25% NHS. After staining with C1q-specific antibodies, cells were analyzed by flow cytometry. **a** and **d** plot the forward scatter (FS) against the sideward scatter (SS). Regions R1 and R2 represent the gates including viable and dead cells, respectively. The histograms compare the binding of C1q-specific antibodies (black line) to the binding of isotype control antibodies (gray line). This comparison is shown in (**e** and **f**) for cells in R1 and R2 separately

**Figure 2:**
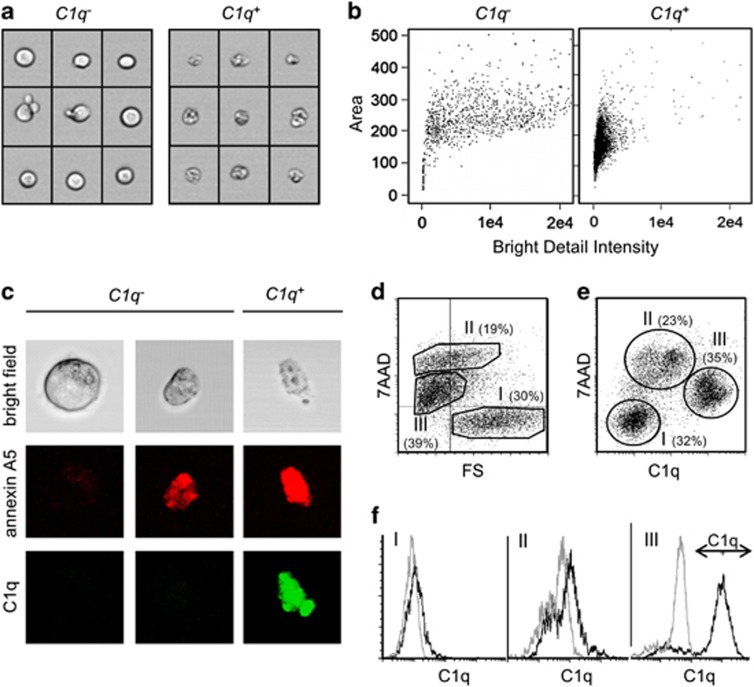
Characterization of C1q-binding cells. Jurkat cells were cultured in UC medium in the presence of 20 *μ*M oxaliplatin for 48 h. Then cells were incubated for an additional hour in UC medium supplemented with 25% NHS. After staining with annexin A5, 7AAD and C1q-specific antibodies cells were analyzed either by imaging flow cytometry (**a** and **b**), confocal microscopy (**c**) or conventional flow cytometry (**d**–**f**). (**a**) Bright field images of representative C1q^−^ and C1q^+^ cells. (**b**) Graphs plotting the area of every image feature (as a measure of the cell volume) against the bright detail intensity (as a measure of the gray value contrast in the bright field image). (**c**) Confocal microscopy images of cells stained with annexin A5 and C1q-specific antibody. (**d** and **e**) Graphs plotting the 7AAD signal against the forward scatter (FS) or against the C1q signal. The indicated percentage values show the distribution of the cells between the three regions I, II and III. (**f**) Comparison of the binding of C1q-specific antibodies (black) with the binding of isotype control antibodies (gray) in the three region I, II and III, respectively (as separated according to the regions shown in **e**)

To characterize C1q binding to dying cells, we incubated viable and apoptotic cells for 1 h in either 20 *μ*g/ml purified C1q or 25% normal human serum (NHS), which contained an equivalent concentration of C1q (Supplementary Figure 3). Untreated control cells had a defined morphology in the FS/SS diagram (region R1 in Figure 1a). About 60% of these cells were C1q^+^ when incubated with purified C1q (Figure 2b). In contrast, after incubation with 25% NHS no binding of C1q could be observed (Figure 2c). Pretreatment with oxaliplatin for 48 h induced apoptotic cell death in about 70% of cells resulting in two populations in the FS/SS plot (Figure 2d): region R1 included still viable cells, whereas region R2 contained smaller dying cells. Purified C1q showed a similar binding affinity to cells in region R1 (Figure 2e) as for untreated cells described above (Figure 2b). Cells in R2, in contrast, showed a much higher C1q binding (see Figure 2e: the mean fluorescence intensity (MFI) was 1959 in R2 and 82 in R1). Incubation of apoptotic cells with NHS (instead of purified C1q) led to a clear and exclusive binding of C1q to apoptotic cells in R2 (Figure 2f). However, this binding was weaker (MFI in R2=254) as with purified C1q. These data suggest that the specificity of C1q for dying cells requires additional serum factors such as C1q-specific inhibitors. Therefore, we considered this situation as more physiologic and focused all subsequent experiments on the effect of NHS. Note that in the presence of NHS, C1q did not bind to about half of the cells in R2, indicating that the population of apoptotic cells was divided in a C1q^+^ and a C1q^−^ subgroup. Primary necrotic cells incubated in NHS, in contrast, showed C1q binding to all cells (Figure 2g).

Next, we used imaging flow cytometry to evaluate the morphology of 20 000 apoptotic cells, which have been incubated for 1 h in NHS and then stained with anti-C1q antibodies (Figures 2a and b). C1q^−^ cells varied in their cell image area and some smaller cells exhibited apoptotic blebs on their surface. C1q^+^ cells showed a similar cell image area as the smaller C1q^−^ cells but their cell border was much more diffused (resulting in a decreased bright detail intensity). This indicates that C1q binds specifically to cells in the later phase of apoptotic cell death. This interpretation could be confirmed in a confocal microscopic analysis of cells treated in the same way and stained with C1q-specific antibodies and annexin A5 (Figure 2c). As expected, large viable cells were negative for both, C1q and annexin A5. Smaller C1q^−^ cells were all positive for annexin A5. C1q^+^ cells showed a similar volume but a frayed cell border, indicating that they are apoptotic cell remnants. Note that apoptotic bodies or blebs, which are formed and released during apoptosis, are much smaller and were not analyzed in this study. The next experiment investigated whether C1q binds specifically to all late apoptotic/secondary necrotic cells. Therefore, cells were treated as above and then stained with 7-aminoactinomycin D (7AAD) and antibodies against C1q. Figure 2d plots the FS as a measure for cell size against the 7AAD signals. Three populations can be observed in this graph. Cells of population I were 7AAD^−^ and showed the largest FS. Cells of population II showed a >100-fold stronger 7AAD signal (7AAD^high^), indicating a complete loss of the cell membrane integrity. These cells were smaller compared with viable cells of population I. Population III included small cells with an intermediate 7AAD signal (7AAD^low^). As apoptotic cells with a permeable membrane are defined as late apoptotic/secondary necrotic, we can conclude that populations II and III represent two subpopulations of this phase of apoptosis. Figure 2e plots 7AAD against C1q of the same experiment. In this plot the same three populations could be identified: (I) 7AAD^−^ cells (97% of these cells were in the corresponding region of Figure 2d), (II) 7AAD^high^ cells (92%) and finally (III) 7AAD^low^ (78%). Only cells of population III were strongly C1q^+^ (Figure 2f). Jurkat cells treated with the other apoptosis inducers resulted in the formation of the same three populations in the C1q/7AAD plot with a similar distribution (Supplementary Figure 4A). Thus, C1q binds in the presence of NHS to a subpopulation of late apoptotic/secondary necrotic cells consisting of dead cell remnants. To evaluate whether these results are cell type specific, we treated cancer cell lines from breast, pancreas and colon (HCC1143, PANC-1 and HT-29) in the same manner with oxaliplatin. All cell lines formed the same three populations as Jurkat cells (Supplementary Figure 3B).

### Effect of C1q binding to late apoptotic cells on efferocytosis

**Figure 3:**
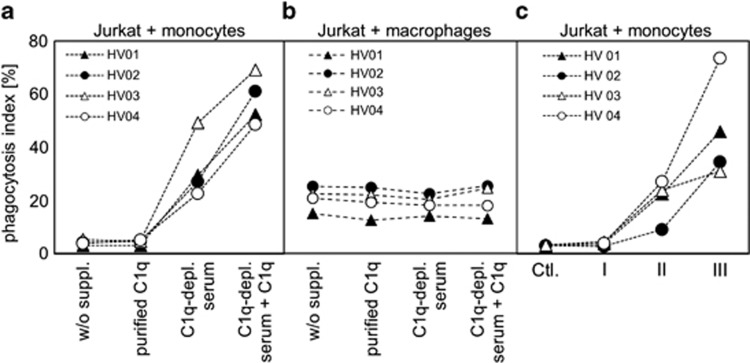
Phagocytosis of dying Jurkat cell. (**a**) Jurkat cells were labeled with CFSE and cultured in UC medium in the presence of 20 *μ*M oxaliplatin for 48 h. Then cells were incubated for an additional hour in UC medium either without supplementation or supplemented with purified C1q, or C1q-depleted serum, or C1q-depleted serum with 20 *μ*g/ml purified C1q. Then cells were mixed with CD14^+^ primary human monocytes from four different volunteers (HV01–HV04) and incubated for an additional 2 h for phagocytosis in serum-free UC medium. (**b**) Jurkat cells were treated the same way as in **a**, then mixed with CD14^+^ monocyte-derived macrophages from four different volunteers (HV01–HV04) and incubated for an additional 2 h for phagocytosis in serum-free UC medium. (**c**) Jurkat cells were treated with oxaliplatin as described above, incubated for 1 h in UC medium supplemented with 25% NHS and then sorted according to the FS/7AAD graph in populations I, II and III. Then the subpopulations were mixed with CD14^+^ primary human monocytes from four different donors (HV01–HV04) and incubated for an additional 2 h for phagocytosis. Untreated cells were used as negative control. The phagocytosis index indicates the percentage of CFSE^+^ monocytes/macrophages as assessed by flow cytometry

Viable Jurkat cells expressed high levels of the ‘do not eat me signal' CD47 on their surface (Supplementary Figure 5) to prevent efferocytosis. Induction of apoptosis decreased this expression independently of the presence of NHS. To investigate the effect of C1q binding to 7AAD^low^ late apoptotic cells on efferocytosis, we labeled apoptotic cells with CFSE and subdivided them into four aliquots. They were incubated for 1 h in UltraCULTURE (UC) medium either without any supplementation (w/o suppl.) or supplemented with purified C1q, C1q-depleted serum or C1q-depleted serum plus purified C1q. Then an equal number of primary human monocytes was added, cocultured for 2 h for phagocytosis in serum-free UC medium and analyzed by flow cytometry (for a microscopic image of the phagocytic process see Supplementary Figure 6). The quantitative analysis of parallel phagocytosis experiments with monocytes from four different healthy volunteers (HV01–HV04) is shown in Figure 3a. Without supplementation we could not observe any substantial phagocytosis of dying Jurkat cells. Preincubation of dying cells with purified C1q did not increase this value. Preincubation with C1q-depleted serum, however, resulted in a phagocytic activity of 30%. Interestingly, this level could be nearly doubled when purified C1q was added during the preincubation. These data illustrate that the strong binding of purified C1q to dying cells shown in Figure 1e has no effect on their elimination by monocytes. An additional exposure to other serum factors is mandatory to get a stimulation of phagocytosis by C1q. Next, we repeated the same experimental setting and cocultured the apoptotic cells with macrophages from four different donors. Macrophages showed a phagocytosis index of around 20% independently of the supplementation (Figure 3b). In a further experiment, apoptotic cells were incubated in NHS for 1 h, stained with 7AAD and sorted by fluorescence-activated cell sorting (FACS) into the populations I, II and III according to the FS/7AAD plot as described above in Figure 2d (note that this approach avoids any potential artificial modification of group III by anti-C1q antibodies). The sorted cells were cocultured with primary human monocytes for 2 h. Almost no phagocytosis could be observed with cells of population I (Figure 3c). Cells of population II were taken up by monocytes to a certain degree. But population III showed a more than twofold higher phagocytosis index. Thus, C1q^+^ dead cell remnants (population III), which are only formed in the presence of NHS, seemed to be most efficiently eliminated by monocytes.

### Cell death progression during late apoptosis

**Figure 4:**
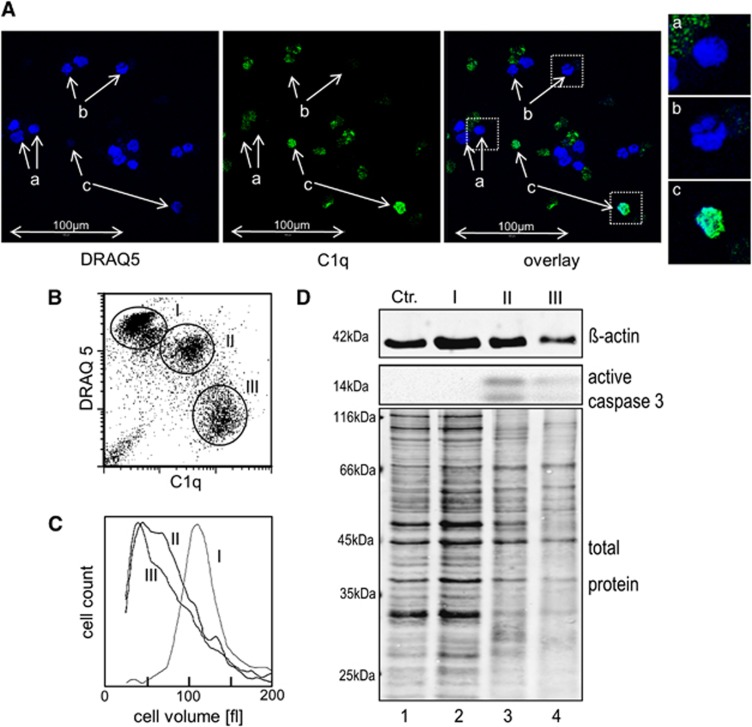
Comparison of late apoptotic subpopulations. (**A** and **B**) DNA content of apoptotic cells. Jurkat cells were oxaliplatin treated for 48 h, incubated for an additional hour in 25% NHS and then stained with C1q-specific antibodies and with the membrane permeable DNA dye DRAQ5. (**A**) Confocal microscopy images show C1q-negative cells with round shaped (a) and fragmented (b) nuclei and C1q-positive cells with almost no DRAQ5 signal (c). (left image, DRAQ5; middle image, C1q; right image, overlay; the three rightmost images show selected cells from overlay). (**B**) The graph plots the C1q signal against the DRAQ5 signal as assessed by flow cytometry. (**C** and **D**) Cell shrinkage and protein degradation during apoptosis. Jurkat cells were oxaliplatin treated and incubated in NHS as described above. Then cells were stained with 7AAD cells and FACS sorted in populations I, II and III according to the FS/7AAD signal. (**C**) Cell volume distribution of the tree populations as determined in an automated cell counter. (**D**) Western blot analysis using anti-*β*-actin antibodies, anti-caspase-3 antibodies and ruthenium-(II)-tris (bathophenanthroline disulfonate) (RuBPS) (for visualization of the total protein)

Oxaliplatin-treated cells were stained for DNA (using DRAQ5) and for C1q and then analyzed by fluorescence microscopy and flow cytometry (Figures 4a and b). DRAQ5-positive cells included those with nicely round shaped nuclei (indicated with a) as well as cells with fragmented nuclei (b). Both cells types were C1q^−^. C1q binding could be only observed in cells with a nearly invisible DRAQ5 signal (c). The cell population with the lowest C1q signal showed a very strong DRAQ5 signal (Figure 4b). DRAQ5 is membrane permeable, and therefore we assume that this signal reflected the normal genomic DNA content of viable cells in population I. The DRAQ5 signal of population II was only slightly reduced pointing to rearrangements in the nuclei during the first phase of late apoptosis. Note that the higher C1q signal of these cells was due to unspecific binding, which could be also observed with isotype control antibodies (see also Figure 2f). The C1q^+^ population III showed a remarkably lower DRAQ5 signal than population II. To quantitate the morphological differences, we FACS sorted the three populations and determined the cell volume (Figure 4c). Populations II and III were both clearly smaller than population I but showed a similar peak volume. This confirms that population III represents cell remnants rather than cell fragments. Western blot analysis of the sorted cells revealed that caspase-3 was strongly activated in populations II and III but not in population I (Figure 4d). Staining the total protein on the western blot membrane indicated remarkable protein degradation in population II compared with population I. This effect was only slightly more pronounced in population III. Taken together, these experiments showed that the most striking difference between the two subpopulations of late apoptotic/secondary necrotic cells is in, besides the different affinity to C1q, the huge difference in the DNA content.

### DNA degradation during late apoptosis is mediated by cell non-autonomous nucleases

**Figure 5:**
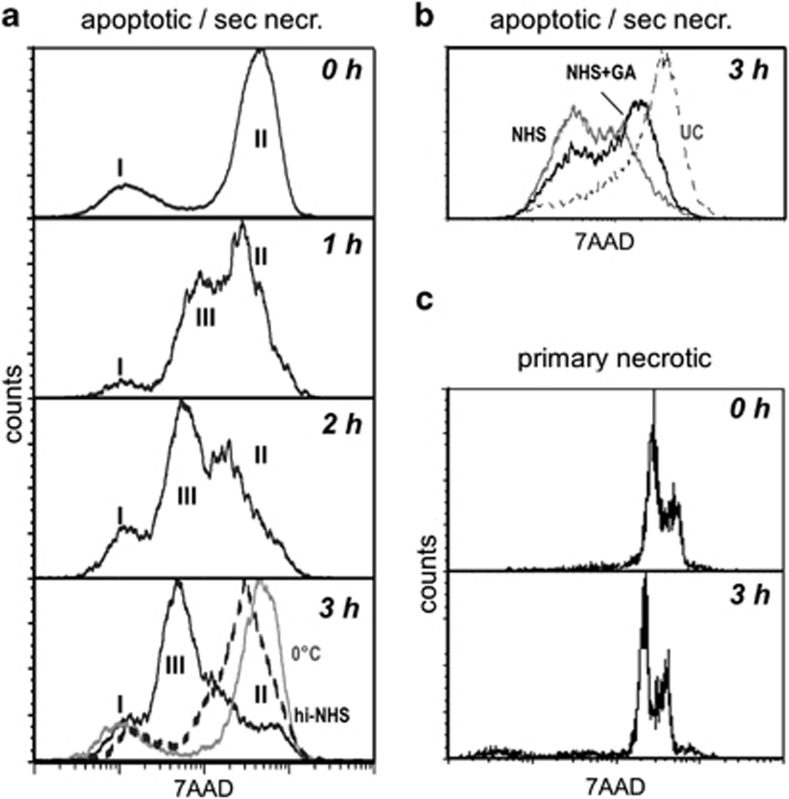
NHS-dependent DNA degradation in apoptotic Jurkat cells. (**a**) Jurkat cells were cultured in UC medium in the presence of 20 μM oxaliplatin for 48 h and stained with 7AAD. Then 25% NHS was added and the 7AAD signal was measured at different points in time (0–3 h). Then the 7AAD signal was measured by flow cytometry. The gray line represents the result for cells incubated at 0 °C. The broken line indicates the result for cells incubated at 37 °C in heat-inactivated NHS. The Roman numerals indicate the position of populations I, II and III. (**b**) Jurkat cells were treated as in **a**, then incubated for 3 h in serum-free UC medium (dotted line), 25% NHS (gray line) or 25% NHS supplemented with the DNase I inhibitor G-actin (black line). (**c**) Jurkat cells were cultured in UC medium, heat shock treated and stained with 7AAD. Then 25% NHS was added and the 7AAD signal was measured at different time points (0–3 h)

Apoptotic cells were stained with 7AAD before any incubation with NHS. The upper most graph in Figure 5a shows that this treatment induced the formation of a 7AAD^high^ population (corresponding to population II in Figure 2d). The remaining 23% of cells were 7AAD^−^ (corresponding to cells of population I). Then cells were incubated in NHS for different times resulting in the formation of an additional peak, which shifted over time to the left (indicated with III). The more detailed Supplementary Figure 7 shows that this peak could already be observed after 45 min of NHS incubation and that this left shift continued over 300 min. The left shift of the 7AAD signal did not occur at 0 °C (gray line) or when cells were incubated with heat-inactivated NHS (broken line). This indicates an NHS-dependent DNA reduction in the late apoptotic cell that is mediated by an enzymatic process rather than simply by a DNA release from the apoptotic cell remnant. Similar experiments with C1q-depleted serum showed a similar decrease indicating that C1q itself is not involved in the DNA degradation (see Supplementary Figure 8). To test whether the NHS-dependent DNA degradation might be related to this plasma DNase I, we repeated the same experiment as above and added 250 *μ*g/ml of the specific inhibitor G-actin. Figure 5b shows that this addition inhibited the NHS-dependent reduction of the 7AAD signal. To investigate whether the NHS-dependent DNA degradation is a general effect after any form of cell membrane damage, we induced primary necrosis. Similar to apoptotic cells, necrotic cells form only one 7AAD^+^ population in the absence of NHS (0 h in Figure 5c; the two peaks of this population probably represent the G1 and G2 phases in the cell cycle). Because of the high efficiency of necrosis induction almost no viable 7AAD^−^ cells could be observed. In contrast to apoptotic cells, necrotic cells did not show any DNA degradation when NHS was added for up to 3 h. Thus, primary necrotic cells react differently in response to NHS addition than late apoptotic/secondary necrotic cells in respect to DNA degradation.

## Discussion

This study revealed that C1q binds to a distinct subpopulation of secondary necrotic cells, which is formed in the presence of serum under involvement of DNase I. This transition is a prerequisite for the stimulatory activity of C1q on efferocytosis.

It is well known that C1q opsonizes dying cells and facilitates their clearance.^7, 8, 17^ The current study revealed that serum-contained inhibitors are required to provide a selective binding of C1q to dying cells. Purified C1q alone binds, albeit weaker, also to viable cells. Martin *et al.*^7^ identified annexin A2 as one of the main binding partners of purified C1q. In accordance to our data, they found that this binding is much weaker in the presence of serum. The strong binding of purified C1q without serum had no effect on efferocytosis (Figure 3). However, the clearly weaker binding of C1q in the presence of serum resulted in a nearly doubled phagocytosis rate. Thus, the capacity of C1q to facilitate efferocytosis is not related to the quantity of bound C1q. On the basis of our results we propose that it is dependent on a serum-mediated processing of late apoptotic/secondary necrotic cells. The selectivity of the efferocytotic process for specific subpopulations of apoptotic cells was observed with monocytes but not with macrophages. This difference indicates that the immune system reacts gradually to cell death. Undifferentiated primary monocytes from the peripheral blood phagocytose preferably C1q^+^ late apoptotic/secondary necrotic cells. Monocyte-derived macrophages, in contrast, seem not to need any dead cell processing for efferocytosis. Dying cells can be eliminated not only by professional phagocytes of the immune system (such as monocytes and macrophages), but also by non-professional phagocytes (such as ‘neighboring' epithelial cells, endothelial cells or fibroblasts).^1^ It has been recently discovered that cells can be also eliminated by cell cannibalism^23^ or entosis.^24^ These processes involve the ingestion of cells, resulting in the unusual appearance of whole cells contained within large vacuoles, or so-called ‘cell-in-cell' structures.^25^ We suspect that the serum-dependent processing of late apoptotic/secondary necrotic cells to small and scraggly C1q^+^ dead cell remnants facilitates ‘non-professional' ways of dead cell clearance. Corroboration for this assumption comes from the observation that ‘non-professional' clearance of dead cells is less efficient than ‘professional' efferocytosis.^26^ Dead cells are rapidly engulfed and cleared by macrophages in mouse embryos. In macrophage-less embryos the task of phagocytosis is taken over by mesenchymal neighbors. But it takes three times as many of these mesenchymal cells to complete the task and they appear to be less efficient than macrophages.

C1q binding occurred exclusively to late apoptotic/secondary necrotic cells undergoing DNA degradation. Already >10 years ago Navratil *et al.*^27^ as well as Nauta *et al.*^28^ mentioned that not all late apoptotic/secondary necrotic cells are opsonized with C1q. However, no further characterization of the C1q-negative population was attempted in this regard. The conversion of 7AAD^high^/C1q^−^ into 7AAD^low^/C1q^+^ cells started immediately after addition of serum. Remarkably, not all cells were susceptible to this degradation and a subpopulation remained 7AAD^high^/C1q^−^ even after 5 h of serum incubation. The DNA breakdown was sensitive to heat inactivation of the serum and to low temperatures. Accordingly, heat-inactivated fetal calf serum (FCS), which is a usual supplement of the cell culture medium, could not induce any DNA degradation (data not shown). This may be the reason why the subdivision of secondary necrotic cells into two populations was not detectable in many studies on apoptosis. The literature defines two classes of nucleases that degrade cellular DNA during apoptosis: (i) cell autonomous nucleases (e.g., caspase activated DNase DFF40 and Endo G), which cleaves DNA within the dying cell and are responsible for DNA laddering and (ii) cell non-autonomous nucleases (e.g., lysosomal DNase II), which derives from the cells that have phagocytosed the apoptotic remnants.^21, 29^ Here, we describe that serum-bound DNase I activity enters the late apoptotic/secondary necrotic cell in the absence of any phagocyte. There is one report on a similar serum-dependent and heat-sensitive DNA degradation in dying cells that has been published by the research group of Martin Herrmann.^17^ The authors proposed that DNase I cooperates with C1q in the clearance of necrotic cell-derived chromatin. However, this effect was very slow. It was detectable after 14 h, whereas we see DNA degradation already after 45 min. In addition, the authors detected the DNA degradation in primary as well as in secondary necrotic cells. In our study, in contrast, we did not observe a DNase activity in primary necrotic cells within 5 h, which points to a distinct mechanism in primary necrotic *versus* late apoptotic/secondary necrotic cells. Although DNA degradation and C1q binding seems to occur simultaneously in secondary necrotic cells, we have no proof that these steps are directly interconnected.

Taken together these results show that serum factors besides C1q are involved in the processing of late apoptotic/secondary necrotic cells promoting the advancement in the cell death progression. The later the step in this progression, the higher was the phagocytosis index in our experiments. Therefore, we propose that the interplay of C1q and its regulators facilitates the detection of an advanced subpopulation of late apoptotic/secondary necrotic cells and promotes a powerful efferocytotic response to remove these cell remnants.

## Materials and Methods

### Materials

The T lymphocyte tumor cell line Jurkat, the breast cancer cell line HCC1143, the pancreatic cancer cell line PANC-1 and colon cancer cell line HT-29 were obtained from ATCC–LGC Standards GmbH, Wesel, Germany. RPMI 1640 medium including GlutaMAX (Invitrogen, Paisley, UK) and DMEM/F12 medium including GlutaMAX (Invitrogen) were supplemented with 10% heat-inactivated FCS (Linaris, Wertheim-Bettingen, Germany). UC medium consisting of serum-free UltraCULTURE (UC) medium (Lonza, Walkersville, MD, USA) supplemented with GlutaMAX (Invitrogen). This medium includes recombinant human insulin, bovine transferrin and purified albumin. Adherent cell lines were detached from culture plates by incubation with trypsin (PAA Laboratories GmbH, Pasching, Austria). Granulocyte macrophage colony-stimulating factor (GM-CSF) was obtained from Berlex (Berlin, Germany). Oxaliplatin, irinotecan, docetaxel, etoposide and 5-fluorouracil were kindly provided by the pharmacy of the General Hospital of Vienna. The EZ4U kit for cell viability was obtained by Biomedica (Vienna, Austria) and analysis was performed on an ELISA reader (Wallac Victor,^3^ PerkinElmer, Waltham, MA, USA). Detection of apoptosis was done by annexin A5 FITC/PI staining (Apoptosis Detection Kit I, 559763, BD Bioscience, San Diego, CA, USA) or annexin A5 PE/7-aminoactinomycin D (7AAD) staining (BD Bioscience). Cell volume was measured using an automated cell counter (Sysmex, Kobe, Japan). NHS was a pool of type AB human sera (AB serum Plus, PAA, Pasching, Austria). C1-depleted human serum was from Quidel, San Diego, CA, USA). Purified C1q protein was obtained from CompTech (Tyler, TX, USA). G-actin from rabbit muscle was obtained by Sigma (St. Louis, MO, USA). Ficoll gradient and CD14-specific magnetic MACS beads for isolation of monocytes were from Miltenyi Biotec (Bergisch Gladbach, Germany). Antibodies used in this study included polyclonal rabbit anti-human C1q antibody (A013602; Dako, Glostrup, Denmark), rabbit negative immunoglobulin control fraction (X0936; Dako), APC-conjugated goat anti-rabbit IgG (X0936; Dako), purified rabbit anti-active caspase-3 (BD pharmingen, Franklin Lakes, NJ, USA), APC-conjugated anti-CD14 antibody (1 : 100; 9017-0149-025; eBioscience, Vienna, Austria), mouse anti-human CD47-FITC (eBioscience, San Diego, CA, USA), rabbit anti-human *ß*-actin polyclonal antibody (Biozol Diagnostica, Eching, Germany), mouse anti-human caspase-3 (Enzo Life Sciences, Farmingdale, NY, USA), mouse anti-caspase-8 (Cell Signaling Technology, Danvers, MA, USA), rabbit anti-human C1q antibody (Dako), Cy5-labeled anti-rabbit IgG antibody (Jackson Immuno Research Laboratories, West Grove, PA, USA) and horseradish peroxidase (HRP)-conjugated anti-mouse IgG antibody (Pierce, Rockford, IL, USA). HRP-conjugated signal was detected with the Supersignal West Femto Detection System (Pierce). Nitrocellulose membranes were scanned using a Typhoon TRIO scanner (GE Healthcare, Uppsala, Sweden). Other dyes include CFSE (Invitrogen, Eugene, OR, USA) and DRAQ5 (1 : 500). After the staining procedure, cells were immediately measured either with a Gallios flow cytometer (Beckman Coulter, Miami, FL, USA) or an image flow cytometer (Image Stream—X Mark II System, Merck Millipore, Darmstadt, Germany). FACS was performed with the FACSAria (BD Biosciences, St Jose, CA, USA) cell sorter. For confocal microscopy special adhesion slides were used. Confocal microscopy images were performed on an LSM 700 laser scanning microscope (Zeiss, Göttingen, Germany).

### Cell lines and culture conditions

Jurkats, HCC1143 and PANC-1 were cultured in RPMI medium. HT-29 cells were cultured in DMEM/F12 medium. All cell lines were cultured at 37 °C and in a 5% CO_2_ atmosphere. To avoid any interference of FCS-derived serum factors, cells were cultured overnight in UC medium before induction of apoptosis. This medium did not induce any reduction in cell viability over a period of several weeks. For flow cytometric analysis adherent cell lines were detached by incubation with trypsin for 10–15 min.

### Induction of cell death

Apoptosis was induced either by incubation of the cell lines with the aforementioned chemotherapeutics for 24–72 h in UC medium or by exposure of cells to 300 mJ/cm^2^ UV-C followed by cell culture in UC medium for 48 h. Titration experiments and time course experiments using an MTT-based assay revealed the drug concentrations that reduced the cell viability by 70% (LD_70_) within 48 h (5-FU 72 h; for further details see Supplementary Figures 1 and 2). Induction of necrosis was performed by heat shock treatment at 58 °C, 20 min in UC medium. Cell viability was determined using the EZ4U assay according to the manufacturer's protocol. Briefly, 200 000 Jurkat cells/well (100 *μ*l) were seeded into 96-well plates in UC medium. The next day chemotherapeutic drugs were added to the cells and the cells were incubated for up to 48 h (5-FU for 72 h) at 37 °C in the CO_2_ incubator. After the incubation time 20 *μ*l dye solution were added to 200 *μ*l sample, further incubated for 2 h at 37 °C and measured at 450 nm using an ELISA reader. In addition, cell death was verified by annexin A5 FITC/PI staining. Cell volume was measured using an automated cell counter.

### Complement binding assay

After incubation with chemotherapeutics for 48 or 72 h, cells were washed twice with PBS and then incubated for (if not otherwise indicated) 1 h at 37 °C in UC medium supplemented with either 25% NHS or 20 *μ*g/ml purified C1q. In some assays C1-depleted human serum or heat-inactivated NHS (1 h at 56–60 °C) was used. Afterward cells were washed in PBS^+/+^ containing 2% BSA and stained with anti-human C1q antibody for 30 min on ice. As isotype control an equal concentration of negative immunoglobulin control fraction was used. For detection, APC-conjugated secondary antibody incubation was performed for 30 min on ice in the dark. Afterward cells were stained with annexin A5-PE and 7AAD. Cells were immediately analyzed by confocal microscopy or flow cytometry either with a Gallios flow cytometer (Beckman Coulter, Brea, CA, USA) or an image flow cytometer. FACS was performed with the FACSAria cell sorter collecting at least 10^6^ cells per population.

### Generation of monocyte-derived macrophages

Monocytes were prepared from healthy individuals by Ficoll gradient centrifugation followed by positive selection using CD14-specific magnetic MACS beads. Cells were cultured in RPMI medium containing GM-CSF for 7–10 days.

### Phagocytosis assay

For the phagocytosis assay, Jurkat cells were labeled for 10 min with 40 *μ*M of CFSE in PBS before induction of apoptosis. As a control, untreated but CFSE-labeled Jurkat cells were used. After 48 h, cells were incubated as described above in UC medium containing 25% of NHS. In the meantime monocytes were prepared as described above. For coculture, 300 000 Jurkat cells were added to 300 000 monocytes and incubated for 2 h at 37 °C in 3 ml UC medium. Afterward cells were washed in PBS^+/+^ containing 2% BSA and monocytes were stained with APC-conjugated anti-CD14 antibody for 30 min on ice. Cells were measured immediately after staining with a Gallios flow cytometer. The distribution of the various cell populations that participate in this phagocytosis assay according to their staining properties is displayed in Supplementary Figure 6. To calculate the phagocytosis index, the cell count of CFSE^+^/CD14^+^ cells was divided by the sum of all CD14^+^ cells and multiplied by the factor 100. As control experiment, cells double positive for CFSE and APC were directly sorted onto special adhesion slides and visualized with an LSM 700 laser scanning microscope.

### Western blotting

Cells were lysed and proteins were extracted as previously described.^30^ For Western blotting experiments, 10 μg per lane of protein extracts were separated on 14% SDS-polyacrylamide gels, transferred onto a nitrocellulose membrane and visualized using ruthenium-(II)-tris (bathophenanthroline disulfonate). Membranes were then incubated either with anti-human *ß*-actin antibody, anti-human caspase-3, anti-caspase-8 or with rabbit anti-human C1q antibody in PBS containing 2% milk and 0.3% Tween-20 for 1.5 h. Bound anti-*ß*-actin or anti-human C1q antibodies were detected with a Cy5-labeled secondary antibody. Bound caspase-3 antibodies were detected with a HRP-conjugated secondary antibody in the presence of Supersignal West Femto Detection System. Nitrocellulose membranes were scanned using a Typhoon TRIO scanner.

## Abbreviations

C1q: complement component C1q
MFI: mean fluorescence intensity
NHS: normal human serum
7AAD: 7-aminoactinomycin D
FACS: fluorescence-activated cell sorting
