# Supplementary figures and images for: Serum-dependent processing of late apoptotic cells for enhanced efferocytosis
Source: Cell Death Dis. 2014 May 29;5(5):e1264–. doi: 10.1038/cddis.2014.210 (PMC4047901; doi:10.1038/cddis.2014.210)

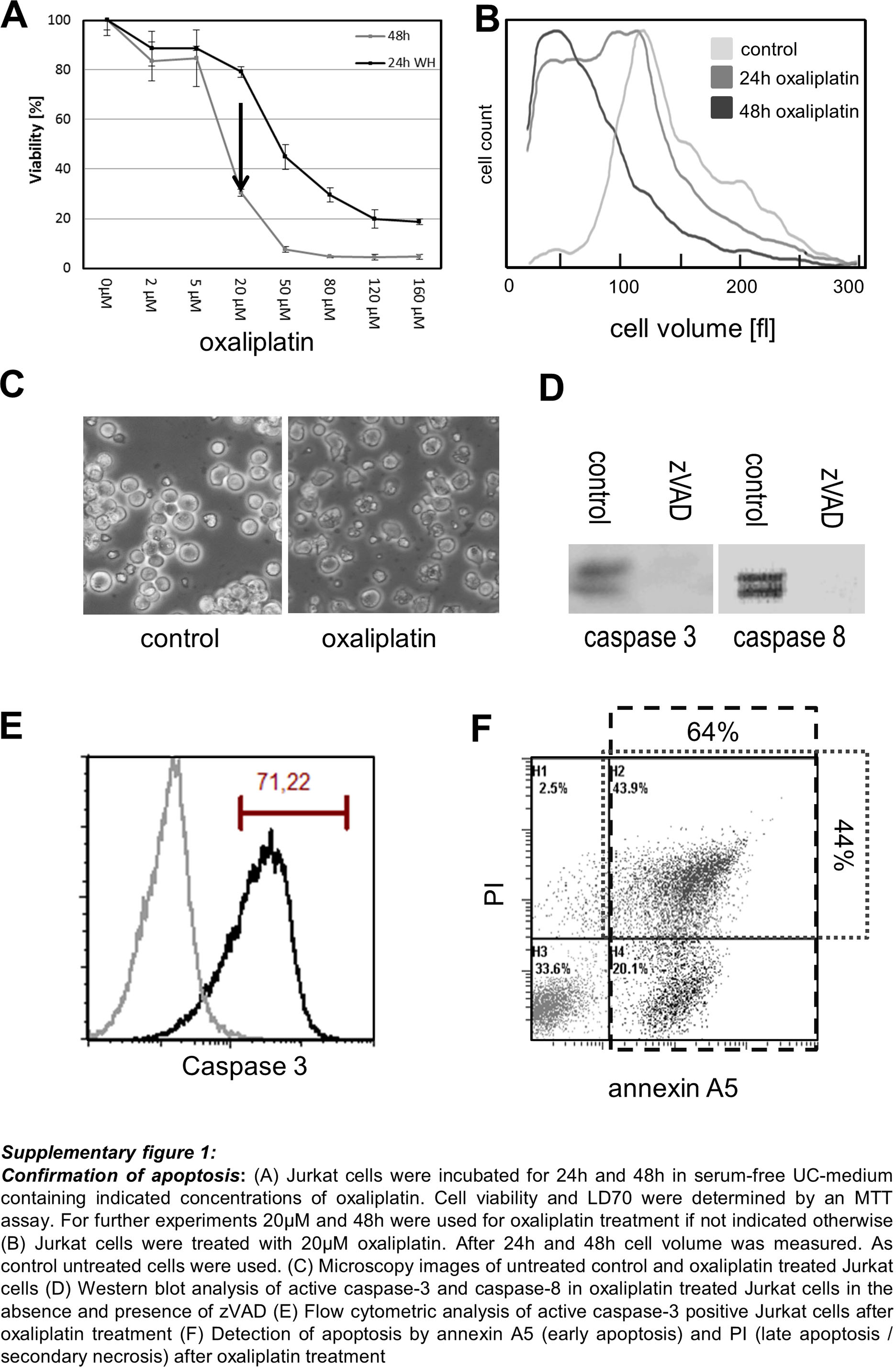

Supplement: Supplementary Figure 1 [file cddis2014210x1.tif]

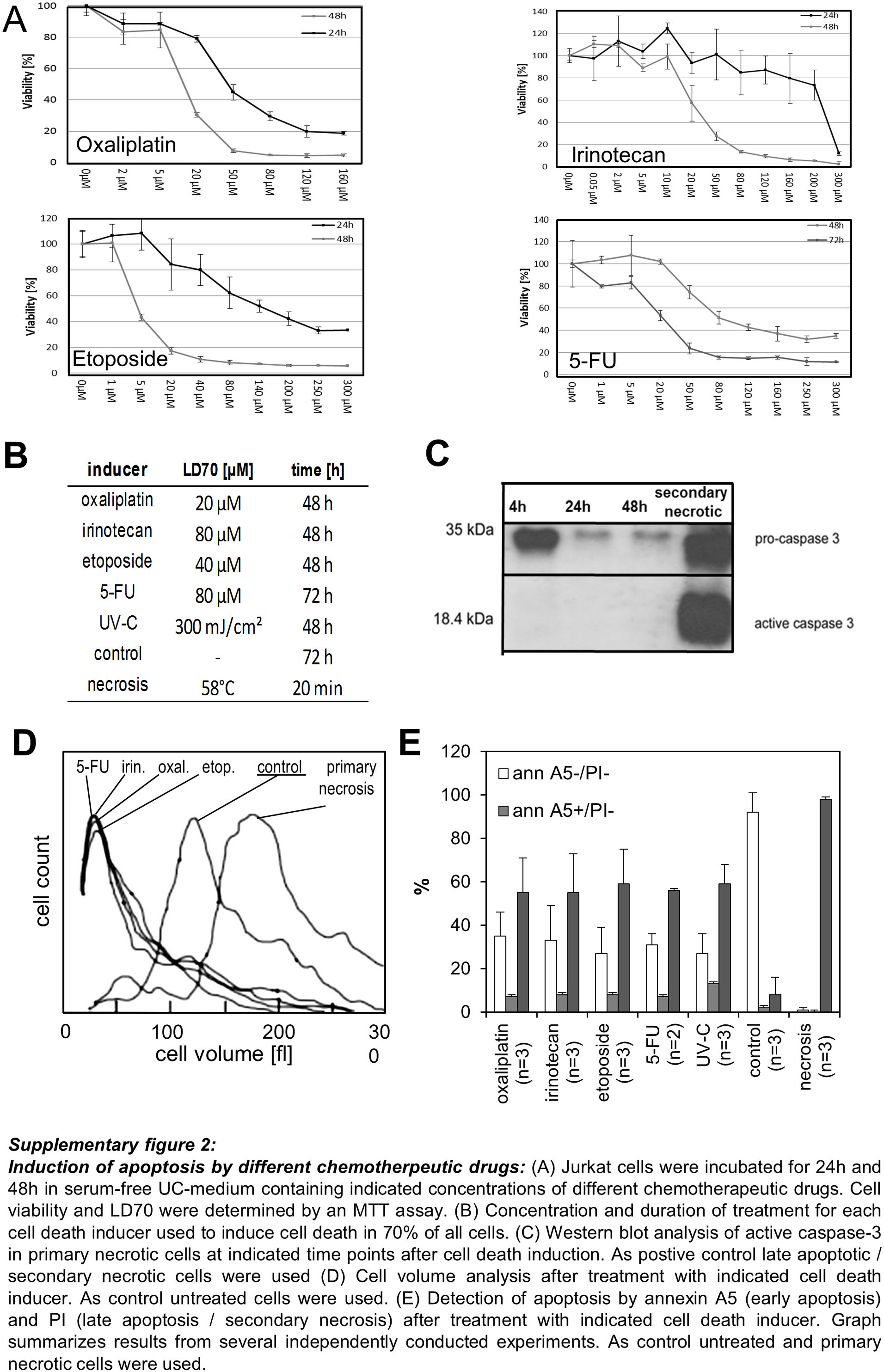

Supplement: Supplementary Figure 2 [file cddis2014210x2.tif]

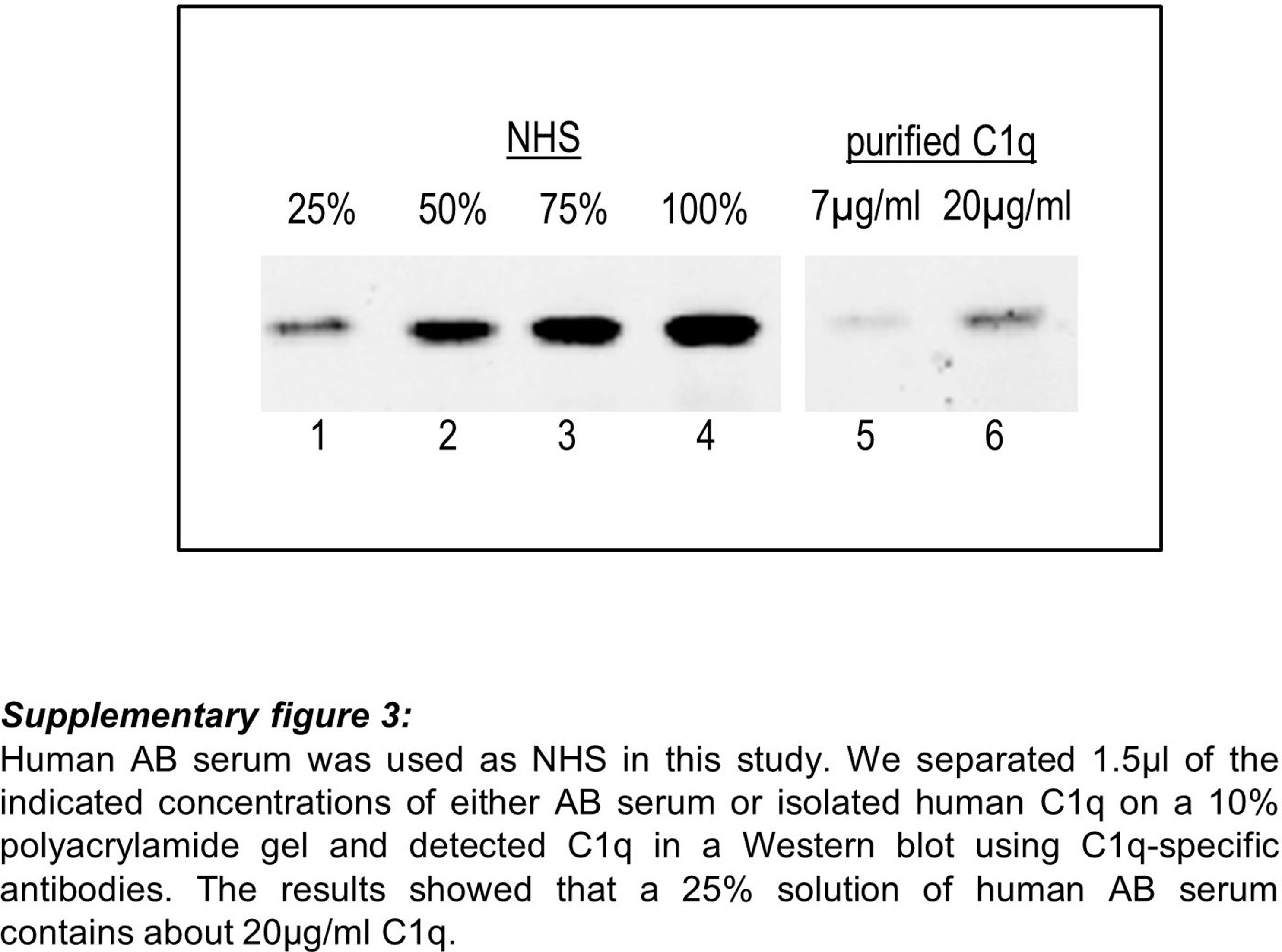

Supplement: Supplementary Figure 3 [file cddis2014210x3.tif]

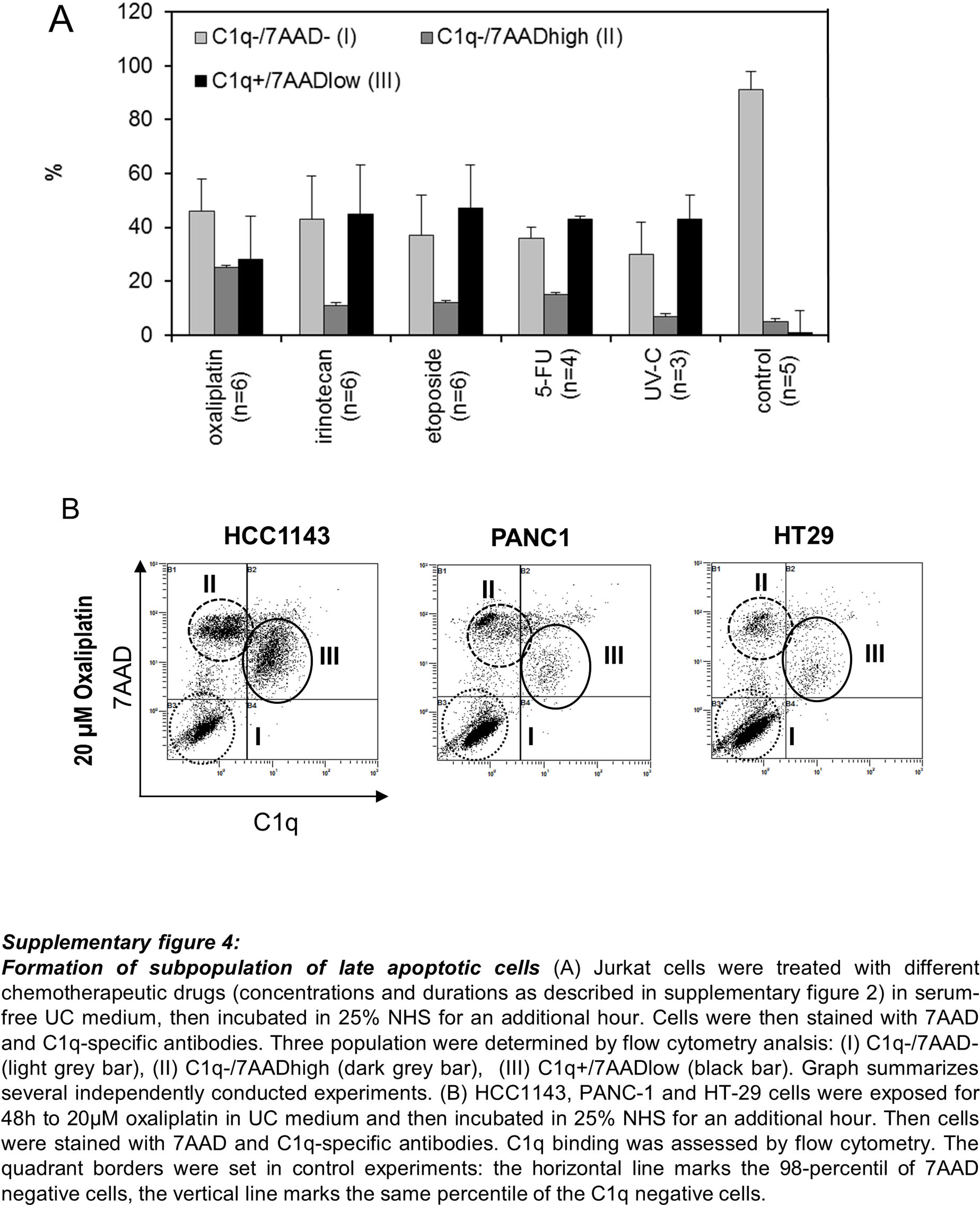

Supplement: Supplementary Figure 4 [file cddis2014210x4.tif]

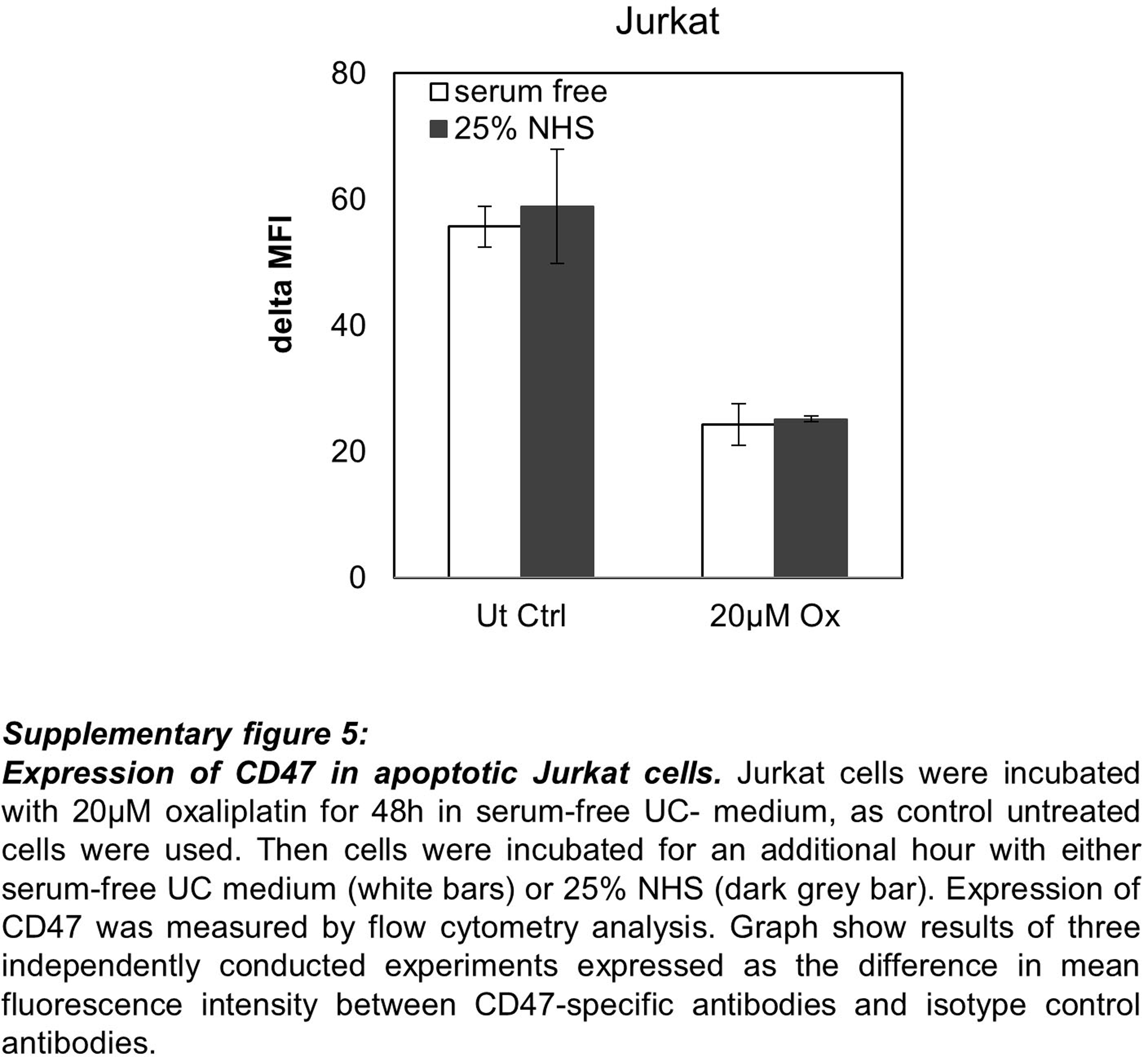

Supplement: Supplementary Figure 5 [file cddis2014210x5.tif]

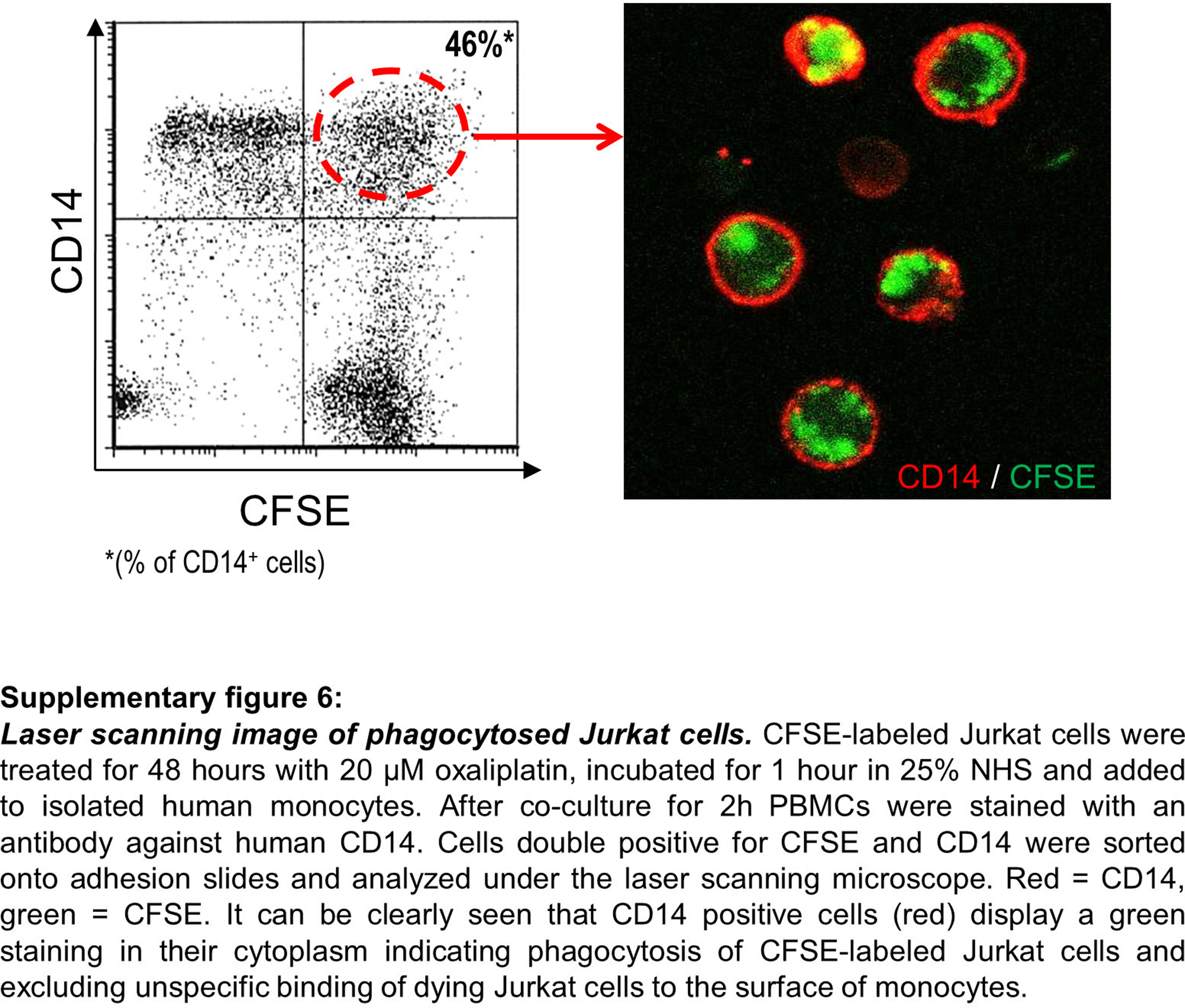

Supplement: Supplementary Figure 6 [file cddis2014210x6.tif]

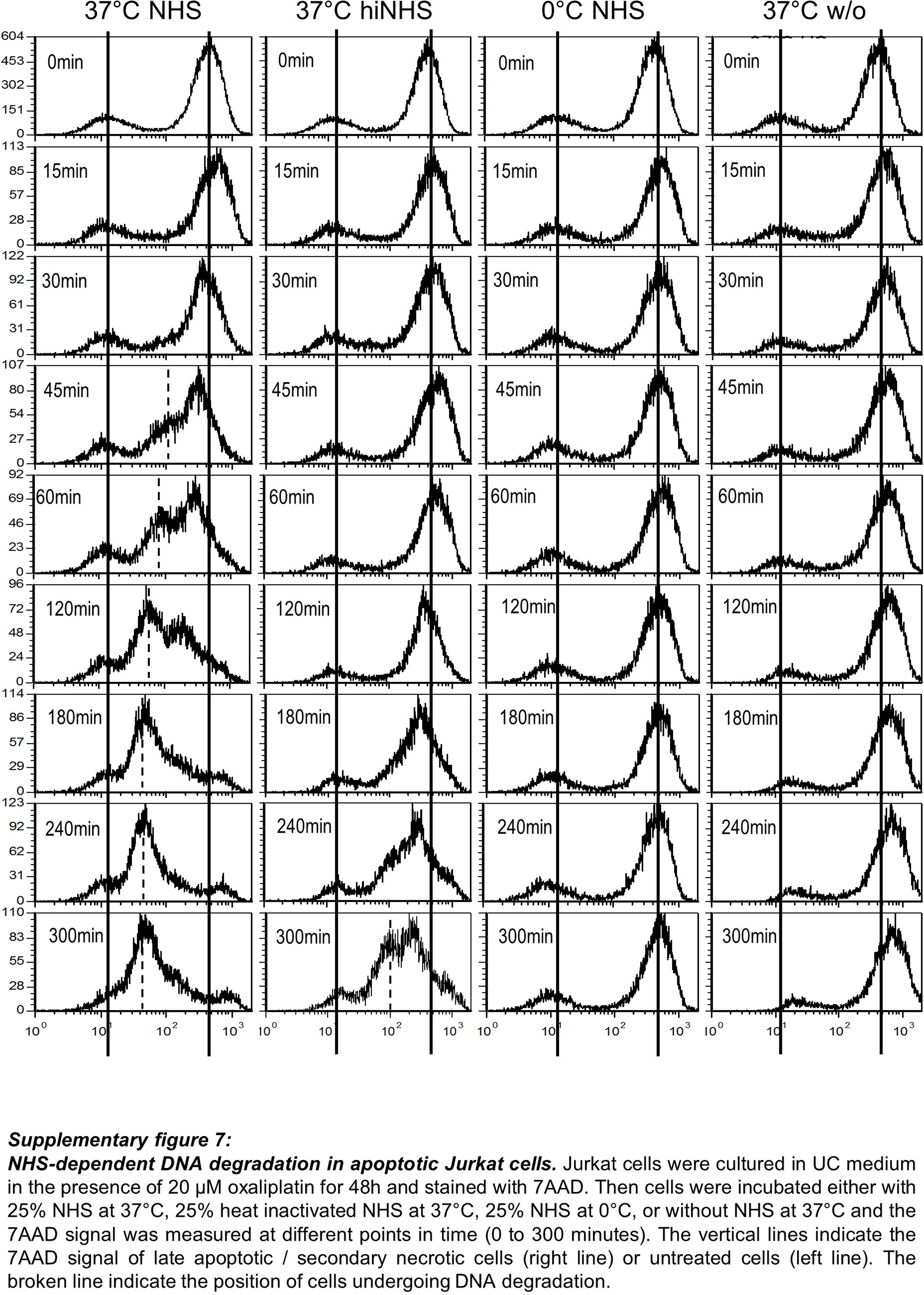

Supplement: Supplementary Figure 7 [file cddis2014210x7.tif]

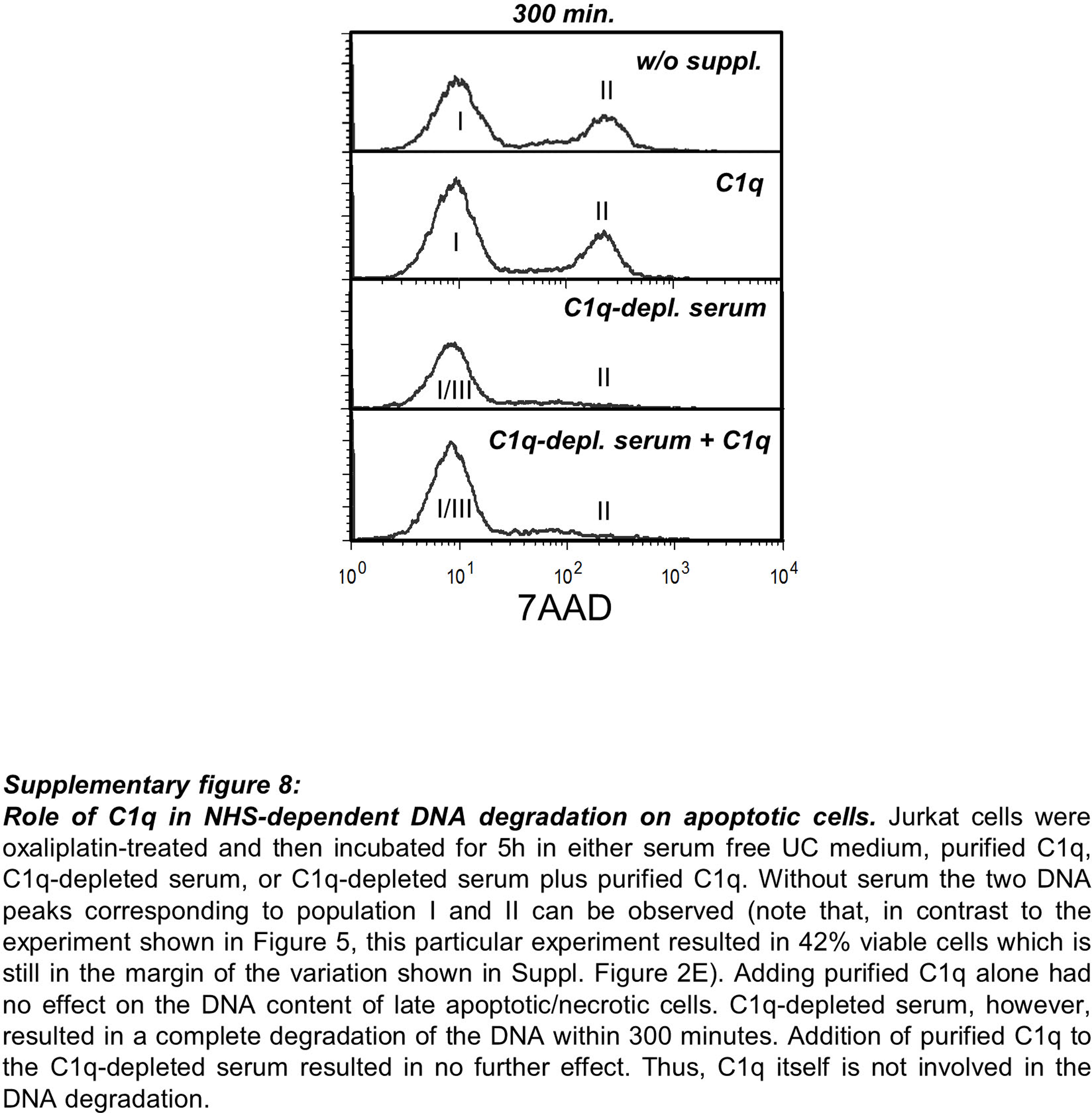

Supplement: Supplementary Figure 8 [file cddis2014210x8.tif]
